# 

**Published:** 1890-01-11

**Authors:** 


### NOTICE TO CORRESPONDENTS.

All MS., Letters, Books for Review, and other matters intended for the Editor, should be addressed The Editor, The Lodge, Porchester Square, London, W.

### Notice to Advertisers and Subscribers.

All Advertisements, Orders for Copies of The Hospital, and Business Communications should be addressed to The Publisher, *not to the Editor*, The Hospital, 139-140, Salisbury-court, Fleet-street, E.C.

### Bound Volumes of "The Hospital."

Vol. VI., containing full page portraits of T.R.H. the Prince and Princess of Wales, and Miss Florence Nightingale, is now ready, 4s., post free. Cases for binding, may be had of the Publisher, at 1s. 6d. each.

### To Residents, Secretaries, and Matrons

The Editor will be glad to have the Regulations and each Report as ssued by Asylums, Hospitals, Convalescent and Nursing Institutions, as well as those of other Charities, and an early copy in duplicate of all Appeals and Festival Notices, etc., addressed to *The Editor, The Lodge, Porchester-square, London, W.* Any superintendent, secretary, matron, or other chief official who regularly sends such information may be registered, on application to the Publisher, to receive free of cost a cover for binding The Hospital in half-yearly volumes.

### Scraps and Gleanings.

A new Danish pharmacopœia is being prepared.

Dundee Royal Infirmary is to be made fire-proof at a cost of £400.

Mr. George Berry is this session delivering the Edinburgh health lectures.

Mr. H. Langdale has been appointed house surgeon to the Oldham Infirmary.

A scheme for the drainage of Cairo is now before the Egyptian Government.

Mr. A. E. Pearson has been appointed resident medical officer to the Leeds Borough Hospital.

The medical stall at the Glasgow bazaar was presided over by doctors' wives dressed as nurses.

The Congress of Internal Medicine will meet at Vienna next year, instead of at Wiesbaden.

The Minnesingers have given a concert at Southsea, which has resulted in £35 given to the hospital.

Lord Ardilaun has given £50 towards the building fund of Dublin Royal Hospital for Incurables.

It is estimated that the electric wires of New York have killed 200 people during the last eight years.

Mr. J. M. Waters has been appointed resident medical officer to the Atkinson Morley Convalescent Home.

Louis H. Sayre, a prominent physician in New York, and son of the famous surgeon, is dead from heart disease.

Mrs. Bateman's dance at Manchester resulted in a sum of £30 given to the Wittington bed of the Ancoats Hospital.

The report of Peterborough Hospital Saturday Fund is out. £331 was received in 1889 against £326 received the previous year.

The Mayor of Hull presided at a public meeting on December 20th, called to consider the financial condition of the infirmary.

The council of the Manchester Jubilee Exhibition have handed their surplus of £43,000 to the Manchester Whitworth Institute.

Mrs. Meredith has cheerful news from the medical missionaries in Palestine; they are very busy, as dengue is there epidemic.

The workpeople's collections for the infirmary at Lancaster this year exceed last year's collections by £268. This is a grand increase.

Dr. Cockburn, of Ulverston, has died suddenly of apoplexy. He was only 39 years of age, and leaves a widow and six young children.

The Princess of Wales gave a donation in aid of Mr. and Mrs. F. T. Davies's fund of £150 for distribution among the St. Pancras poor.

Consular reports received at Vienna state that the cholera is still raging at Hamadan, and that the number of fatal cases is considerable.

Worcester Infirmary propose to transfer part of its capital from Consols to the Town Corporation Stocks. This will increase the income £108 a year.

The annual meeting of Sevenoaks Holmesdale Cottage Hospital was held on December 19th, Earl Stanhope presiding. The report was most satisfactory.

At a drawing-room meeting at Mrs. Welch's, Manchester, £12 was collected for the Mission to Deep-Sea Fishermen. This is a capital way of raising money.

Professor Lawson Tait, Dr. Alex Bowie, and Mr. Berdoe (better known as Æsculapius Scalpel) have signified their approval of the Shaftesbury Hospital.

The Medico-Ethical Society of Bradford has decided that the out-patients to the infirmary cannot be asked to pay a certain sum so long as the medical men give their services gratuitously.

Lord George Hamilton will take the chair at the sixty-ninth annual court of governors of the Dreadnought Seamen's Hospital, at the Hotel Metropole, on Tuesday, the 18th February, at three p.m.

Edinburgh Infirmary has received £144 from the inhabitants of the Orkney Isles—the *ultima Thule* of Great Britain. Considering the poverty of these poor fisher folk, such generosity is most commendable.

Sir Robert Rawlinson's paper on "London Sewage" will be discussed at the Society of Arts on January 15th. Mr. Brudenell Carter will lecture on "Vision-testing for Practical Purposes" on January 22nd.

The working-men supporters of Bolton Infirmary have held their quarterly meeting. Some of the working-men visit the infirmary every Saturday afternoon, and they profess themselves pleased with what they see.

Prince Albert Victor has performed the ceremony of opening the Rangoon Drainage and High-Pressure Water Supply Works, which are being constructed on the Shone system, and are the first of the kind opened in India.

Miss Helen Webb, M.B.Lond., L.S.A., a student of the London School of Medicine for Women, has been appointed assistant medical officer under the Metropolitan Asylums Board. It is the first time a lady has received such a post.

The Vicar of St. Augustine's, Highbury, after the annual collection for the poor of Islington (of which Highbury New Park is a part), said there was not a single poor person in his parish to claim upon the offertory or parochial organisations.

The appearance of the fifteenth thousand of "Science Lectures for the People" is a convincing proof that the people know how to appreciate the efforts of Professor Huxley, Sir Henry Roscoe, the Rev. W. H. Dallinger, and other scientific authorities both to interest and instruct them.

The Prince of Wales will, in the coming spring, officiate at many charity functions if his health permits. He will take the chair at a dinner at the Hotel Metropole on January 13, in aid of a National Leprosy Fund. His Royal Highness's name is also mentioned in connection with dinners for the Royal Free, Royal London Ophthalmic Hospitals, and other charities.

